# Cholinergic urticaria following active forced-air warming in the post-anesthesia care unit: a case report and literature review

**DOI:** 10.3389/fmed.2026.1965980

**Published:** 2026-09-29

**Authors:** Xin Zhou, Mengyu Zhang, Ping Zheng

**Affiliations:** 1 Department of Anesthesiology, West China Tianfu Hospital, Sichuan University, Chengdu, Sichuan, China; 2 Department of Anesthesiology, West China Hospital, Sichuan University/ West China School of Nursing, Sichuan University, Chengdu, Sichuan, China

**Keywords:** active forced-air warming, cholinergic urticaria, differential diagnosis, post-anesthesia recovery period, temperature management

## Abstract

**Introduction:**

Cholinergic urticaria (CU), a subtype of chronic inducible urticaria, is triggered by elevations in core body temperature. Postoperative shivering and active warming measures may increase core body temperature in the post-anesthesia care unit (PACU), yet CU in this setting remains underrecognized and has rarely been reported.

**Case presentation:**

We report a case of an 18-year-old male who developed acute CU in the PACU following cochlear implantation under general anesthesia. After extubation, the patient exhibited postoperative shivering and was treated with a forced-air warming blanket. Approximately 10 min later, he developed diffuse punctate wheals with pale centers and peripheral erythematous halos across the trunk, accompanied by severe pruritus and tachycardia (110–126 beats/min). Given the patient's history of exercise- and heat-induced eruptions, together with stable hemodynamics and the absence of respiratory distress, the clinical findings were considered more consistent with cholinergic urticaria than with severe perioperative hypersensitivity.

**Results:**

Immediate cessation of active warming, intravenous diphenhydramine (20 mg), and environmental cooling resulted in rapid symptom relief within 20 min.

**Conclusion:**

This case underscores the synergistic pathophysiology of postoperative shivering, active warming, and extubation-related sympathetic activation in precipitating CU. This case suggests that postoperative shivering, active warming, and extubation-related sympathetic activation occurred in close temporal sequence with the CU episode and may have jointly contributed to the rapid rise in core temperature and symptom onset. CU may occur during post-anesthesia recovery and should be promptly recognized on the basis of rash morphology, vital signs, and dynamic body temperature changes, differentiated from perioperative anaphylaxis, and managed with targeted temperature control and symptomatic treatment.

## 1 Introduction

Cholinergic urticaria (CU), a subtype of chronic inducible urticaria, was first described by Duke (1) in 1924. Its characteristic clinical presentation involves the development of wheals, predominantly on the trunk and proximal upper extremities, following an increase in core body temperature triggered by exercise, hot baths, or emotional stress. Although the precise pathogenesis remains incompletely elucidated, current evidence supports a multifactorial etiology encompassing histamine release from mast cells, hypersensitivity to sweat components, cholinergic neurotransmitter dysregulation, alterations in serum factors, and anhidrosis or hypohidrosis (2, 3).

The post - anesthesia recovery period represents a critical phase characterized by substantial physiological perturbations in surgical patients. As the inhibitory effects of general anesthetics on the hypothalamic thermoregulatory center gradually subside, patients may experience thermoregulatory disturbances (4). Recovery of consciousness, postoperative shivering, active warming, and extubation-related stimulation may collectively promote an increase in core temperature (5, 6). As anesthetic depth decreases and sympathetic nervous system activity recovers, responsiveness to noxious stimuli also increases (6). Together, these factors may provide a potential physiological context for CU episodes in the PACU.

However, during this phase, clinical manifestations that mimic cholinergic urticaria frequently occur secondary to residual anesthetic agents, surgical trauma, or transfusion and infusion reactions, thus complicating both diagnostic reasoning and clinical treatment. Currently, there is a paucity of literature regarding cholinergic urticaria in the post-anesthesia period, along with a lack of systematic evaluation and management guidelines. This article present the clinical data of a patient who developed cholinergic urticaria during the post-anesthesia recovery period. Through a concurrent literature review, we examined the triggering factors, differential diagnosis, and diagnostic and therapeutic strategies to enhance clinicians' ability to recognize and manage this condition. This case report was prepared in accordance with the CARE guideline.

## 2 Case presentation

An 18-year-old male patient (height 177 cm, weight 66 kg, American Society of Anesthesiologists physical status II) was scheduled to undergo cochlear implantation and inner ear window membrane repair for bilateral profound sensorineural hearing loss. He had no history of drug or food allergies. His preoperative body temperature was 36.5 °C. Notably, a detailed preoperative interview revealed that the patient had experienced self-limiting pruritic rashes after strenuous exercise or hot baths in the past, although there was no formal medical diagnosis.

Vital signs on admission to the operating room were as follows: body temperature 36.4℃, heart rate 62 bpm, blood pressure 126/68 mmHg, and SpO₂98%. A routine anesthetic preparation was carried out. General anesthesia was induced sequentially with intravenous midazolam (2 mg), propofol (130 mg), sufentanil (17.5 µg), and rocuronium (35 mg). Tracheal intubation was performed after confirming adequate neuromuscular blockade. Anesthesia was maintained with inhaled sevoflurane (end-tidal concentration 1.0–1.5 MAC) in an oxygen-air mixture, supplemented by intermittent boluses of analgesics and muscle relaxants. Since the surgical field was confined to the head with only the auricular region exposed, the remainder of the body was covered with bedding and sterile drapes. No active warming measures were used intraoperatively. The surgical procedure proceeded uneventfully, and the operative time was 264 min. Core temperature was continuously monitored using the iThermonitor WT701 (Raiing Medical, Boston, MA), a wireless thermometer attached to the axillary skin surface. Although positioned at the axilla, this device has been validated against distal esophageal core temperature in adults undergoing noncardiac surgery, showing good agreement (mean bias 0.14 °C; Bland–Altman 95% limits of agreement ±0.52 °C; 91% of measurements within ±0.5 °C) (7). In addition, our institution previously validated this device in adults undergoing noncardiac surgery in 2023, with similar findings. The axillary temperature recorded by the WT701 showed good agreement with the pharyngeal reference core temperature (mean bias −0.06 °C ± 0.25 °C). The Bland–Altman 95% limits of agreement for the iThermonitor were relatively narrow, with an estimated upper limit of 0.37 °C (95% CI, 0.30–0.45) and a lower limit of −0.73 °C (95% CI, −0.81 to −0.66). Therefore, the iThermonitor WT701 is considered a suitable surrogate for core temperature monitoring. Intraoperative vital signs remained stable, except that the core body temperature gradually decreased to a nadir of 36.1 °C. The patient was subsequently transferred to the post - anesthesia care unit (PACU) for extubation and recovery. In our institution, patients who have not fully met the emergence and extubation criteria at the end of surgery are routinely transferred to the PACU with the endotracheal tube in place and then extubated after comprehensive assessment.

Fifteen minutes after PACU admission, the patient's consciousness and spontaneous respiration returned, fulfilling standard extubation criteria, and the endotracheal tube was uneventfully removed. Immediately after extubation, the patient developed marked shivering. At that time, his body temperature was 36.1 °C. An active forced - air warming device was applied to maintain his body temperature. Approximately 10 min later, the patient became agitated and reported severe pruritus on his upper trunk. Physical examination revealed the upper trunk covered with confluent, punctate-to-patchy urticarial wheals (1–3 mm in diameter), characterized by pale centers and peripheral erythematous halos (Figure 1). His vital signs showed tachycardia (110–126 bpm), blood pressure of 120/75 mmHg, respiratory rate of 18 bpm, and SpO₂ of 98% while breathing room air (FiO₂ 0.21). Bedside pulmonary auscultation revealed clear bilateral breath sounds, with no wheezing or signs of respiratory distress. His body temperature had risen to 36.8 °C (*Δ*T + 0.7 °C from PACU admission).

**Figure 1:**
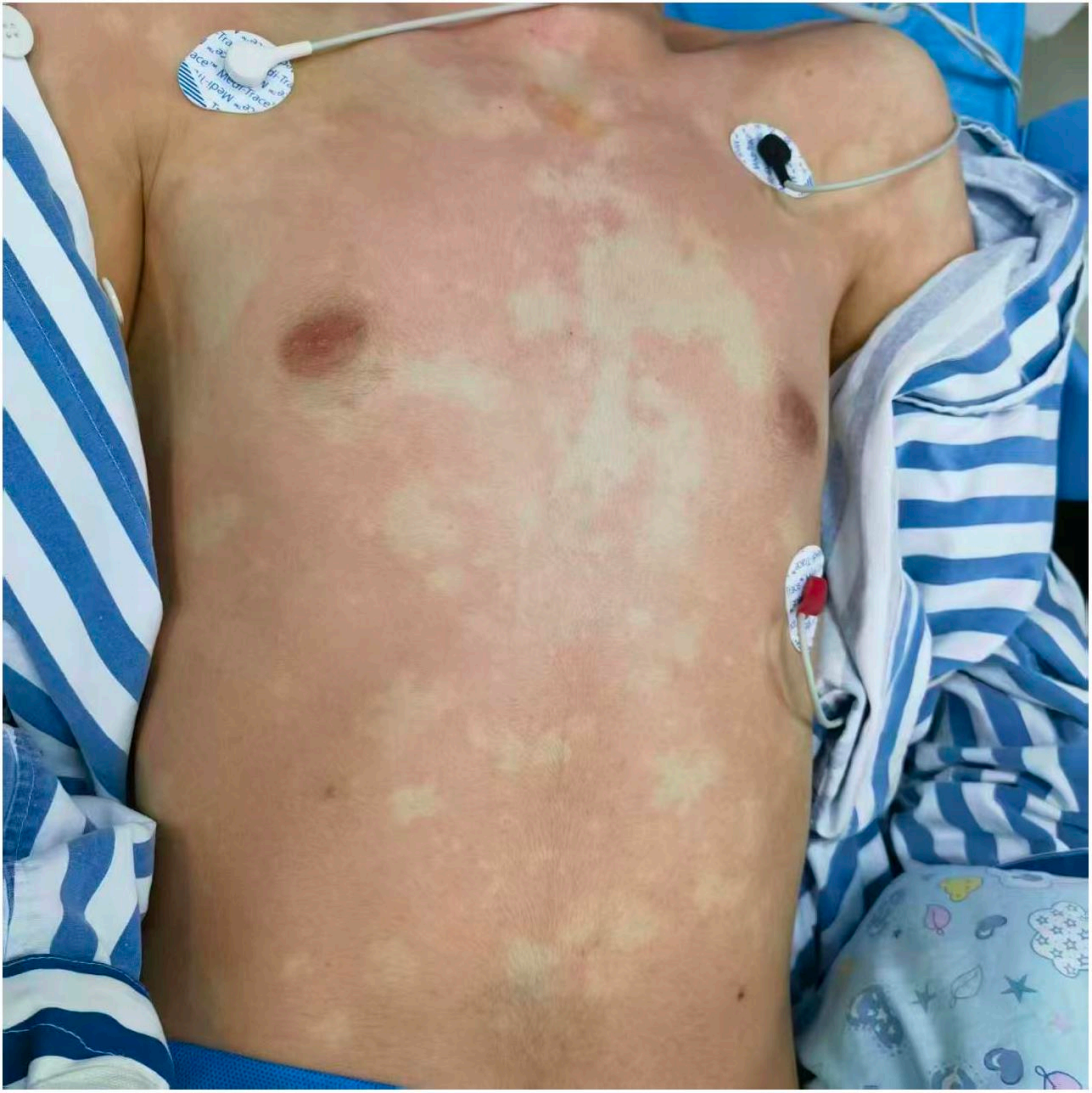
Clinical photograph of the patient's trunk, showing diffuse, confluent, punctate-to-patchy urticarial wheals (1–3 mm) with pale centers and surrounding erythematous halos on the upper trunk, consistent with cholinergic urticaria.

Based on the temporal relationship between the onset of rash and rapid temperature elevation, the clinical manifestations strictly confined to the skin, and crucially, the absence of hypotension or bronchospasm, the likelihood of severe perioperative anaphylaxis was relatively low. Combined with the patient's medical history of rash induced by exercise and heat exposure, cholinergic urticaria is highly suspected after comprehensive evaluation of clinical manifestations. Multiple interventions were implemented: the forced-air warming device was promptly discontinued, and the ambient temperature in the PACU was lowered to 21 ℃ to prevent further body temperature elevation. Intravenous diphenhydramine hydrochloride (20 mg) was administered as an antihistamine. Non-pharmacological measures, including reassurance and written communication adapted to the patient's hearing impairment, were employed to calm the patient and alleviate anxiety and agitation. No additional benzodiazepines or other sedative medications were administered during the episode. Twenty minutes later, the patient reported a significant reduction in pruritus. Physical examination revealed that the rash was fading without the emergence of new wheals, and the heart rate was gradually returning to baseline. After 30 min of continued monitoring in the PACU, the rash had largely subsided, and the patient's vital signs remained stable. The patient was then transferred to the ward. A 24-hour postoperative follow-up revealed no recurrence of pruritus or rash. The patient was discharged on postoperative day 3 after a 7-day hospital stay (including 3 days of preoperative evaluation).

**Patient perspective:** Because of his hearing impairment, the patient communicated mainly in writing. During the episode, he reported intense pruritus; Twenty minutes after treatment, he reported that the pruritus had been significantly relieved.

The perioperative course and key clinical findings were summarized in Table 1.

**Table 1:** Timeline of the clinical course and key events.

Time point	Clinical course and key events
Preoperative assessment	Body temperature was 36.5 °C; there was no history of allergies.
On admission to the operating room	Body temperature was 36.4℃; HR 62 bpm; BP 126/68 mmHg; SpO₂ 98%.
Intraoperative period	The lowest measured body temperature was 36.1 °C; no active warming measures were employed.
Immediately after extubation in the PACU	Marked shivering was observed; body temperature was 36.1 °C; forced-air warming was initiated.
Approximately 10 min after forced-air warming was initiated	Urticarial eruption with severe pruritus; body temperature 36.8 °C; HR 110–126 bpm; BP 120/75 mmHg; RR 18 bpm; SpO₂ 98%.
After treatment	Forced-air warming was discontinued, the PACU ambient temperature was lowered to 21℃, and intravenous diphenhydramine 20 mg was administered; the symptoms were markedly relieved within 20 min.
Follow-up	No recurrence of pruritus or rash was observed during the postoperative period; the patient was discharged on postoperative day 3, with a total hospital stay of 7 days (including 3 days of preoperative evaluation).

## 3 Discussion

The post - anesthesia recovery period is a critical phase characterized by dramatic fluctuations in thermoregulation and autonomic nervous system function. It represents a time of heightened susceptibility to perioperative complications. This case highlights an often - overlooked, non - pharmacologic etiology of acute urticaria in the PACU and underscores the imperative for systematic differential diagnosis and optimized perioperative management. In retrospect, the patient's preoperative history of recurrent pruritic eruptions after exercise and hot baths was highly suggestive of cholinergic urticaria and therefore warranted particular attention during perioperative planning. If cholinergic urticaria had been confirmed preoperatively, continuation of established second-generation H_1_-antihistamine therapy, avoidance of known thermal and emotional triggers, and individualized perioperative temperature management could reasonably have been considered (3, 8). Because emotional stress is a recognized trigger of cholinergic urticaria, appropriate perioperative anxiolysis may theoretically reduce this potential trigger. However, evidence is insufficient to recommend a specific anxiolytic drug or regimen solely for the prevention of perioperative CU, and such management should be individualized (3, 8). Evidence is also currently insufficient to support routine additional intraoperative prophylactic antihistamine administration beyond continuation of established therapy specifically for perioperative CU prevention (8, 9). These considerations further highlight the importance of recognizing potential perioperative triggers of cholinergic urticaria.

General anesthesia impairs normal thermoregulation and promotes redistribution of heat from the core to the peripheral compartment, thereby increasing the risk of perioperative hypothermia (10). In patients with known cholinergic urticaria, perioperative temperature management should balance the prevention of hypothermia with the avoidance of rapid or excessive warming that may provoke sweating or abrupt increases in temperature. Temperature should therefore be closely monitored, and active warming should be titrated to maintain normothermia rather than applied aggressively.

Cholinergic urticaria is more commonly triggered by an increase in core temperature relative to an individual's baseline rather than by crossing a fixed absolute temperature threshold; exercise, heat exposure, and emotional stress can all produce such dynamic increases (2, 3, 10). Thus, in this case, the dynamic increase in temperature from 36.1 °C to 36.8 °C in the PACU (*Δ*T + 0.7 °C), rather than an absolute threshold of 36 °C, was considered more clinically relevant. CU typically manifests when patients are fully emerging from general anesthesia. The mechanism of CU during the post-anesthesia recovery period is unique and complex. In this case, postoperative shivering, active warming, and extubation-related stimulation occurred in close temporal succession and may have jointly contributed to the rise in core temperature and the onset of symptoms. First, post-anesthetic shivering is a sympathetic-mediated thermogenic response that can augment metabolic rate by up to 200%–500% (5). Second, the coexistence of external active warming and endogenous heat production may have accelerated the increase in core temperature (11). Third, the intense stimulation of tracheal extubation restores sympathetic tone and triggers substantial catecholamine release, further accelerating metabolism and elevating core body temperature (12). The convergence of these factors caused a sharp, acute elevation in core body temperature. This abrupt thermal spike stimulated cholinergic nerve endings to release acetylcholine. Acetylcholine then binds to receptors on the mast-cell surface. This interaction precipitated mast cell degranulation and the cascade release of pro-inflammatory mediators—predominantly histamine, leukotrienes, and prostaglandins (3). Eventually, the characteristic cutaneous manifestations emerged. Once CU was recognized, the immediate therapeutic priority was cessation of further core temperature elevation. To facilitate heat dissipation, the forced-air warming device was discontinued, the PACU ambient temperature was lowered to 20–22 °C, and the patient was uncovered. After 20 min, the patient reported relief.

When patients present with acute urticaria in the PACU, clinicians typically suspect drug allergies, anaphylactoid reactions, or transfusion reactions (13); therefore, accurate differential diagnosis is crucial for implementing appropriate therapeutic interventions. Based on a literature review and this case presentation, cholinergic urticaria typically presents as small, punctate papular wheals (1–4 mm in diameter) characterized by pale centers and red halos, often exhibiting a classic “satellite-like” pattern (14). The rash is diffusely distributed, predominantly on the upper trunk, neck, and face (15). It is frequently accompanied by tachycardia due to histamine release and elevated body temperature. Critically, hemodynamic stability is maintained, and life-threatening features of anaphylaxis, such as hypotension, bronchospasm, and laryngeal edema, are notably absent (16). In order to facilitate rapid clinical judgment, Table 2 summarizes the main differential diagnoses of acute urticaria-like reactions in the PACU (13–21).

**Table 2:** Main differential diagnoses of acute postoperative urticaria-like reactions in the PACU.

Differential diagnosis	Characteristic clinical manifestations	Common onset clues/timing	Hemodynamic and respiratory manifestations	Key management principles
Cholinergic urticaria	Small punctate or papular wheals (1–4 mm) with central pallor and surrounding erythematous flare, often accompanied by marked pruritus	Temporally associated with exercise, heat exposure, emotional stress, or a dynamic rise in core body temperature	Hemodynamics are usually stable; hypotension, bronchospasm, and laryngeal edema are typically absent	Discontinue or reduce thermal stimuli and facilitate heat dissipation; provide symptomatic treatment with antihistamines and continue close observation
Perioperative drug hypersensitivity/anaphylaxis	Urticaria and flushing, sometimes accompanied by angioedema; severe cases may involve multiple organ systems	Often temporally related to exposure to antibiotics, neuromuscular blocking agents, or other perioperative medications, and may occur rapidly after drug administration	Hypotension, bronchospasm, hypoxemia, or circulatory instability may occur	Immediately discontinue the suspected agent; manage the airway and circulation and administer epinephrine as indicated for anaphylaxis management
Opioid-related non–immune-mediated histamine release	Pruritus, flushing, or urticaria-like eruptions that may be associated with opioid administration	Usually temporally related to opioid administration	Predominantly cutaneous manifestations; severe circulatory or respiratory involvement is relatively uncommon, although true allergic reactions must still be considered	Assess the temporal relationship to drug administration and the presence of systemic involvement; discontinue the suspected agent and provide symptomatic treatment
Transfusion/blood product–related allergic reaction	Urticaria, pruritus, and flushing; severe reactions may be accompanied by respiratory or circulatory abnormalities	Occurs during or shortly after transfusion or exposure to blood products	Hemodynamics may remain stable in mild cases; anaphylaxis may present with hypotension, wheezing, or hypoxemia	Immediately stop the transfusion, assess reaction severity, and manage according to established transfusion reaction protocols

In the postoperative period, drug - induced hypersensitivity reactions, particularly those mediated by the use of antibiotics and neuromuscular blocking agents, must first be excluded. This is because they usually emerge either before or after anesthesia induction (17, 18). Moreover, anaphylaxis can initiate a general reaction. In contrast, cholinergic urticaria typically manifests as a localized cutaneous reaction with rare systemic involvement (19). In the present case, the rash appeared after a rapid rise in body temperature. At the same time, the blood pressure remained stable, no respiratory symptoms were observed, and the patient had a history of attacks after exercise and bathing. These characteristics generally supported cholinergic urticaria and reduced the possibility of perioperative anaphylaxis.

As soon as cholinergic urticaria is confirmed or strongly suspected, targeted pharmacotherapy should be initiated promptly. According to the EAACI/GA^2^LEN/EDF/WAO guidelines, second-generation H_1_ antihistamines constitute first-line therapy for chronic inducible urticaria (22). In this case, intravenous diphenhydramine (20 mg) was administered for symptomatic treatment. Its use in this closely monitored postoperative setting should not be interpreted as suggesting that diphenhydramine is preferred over second-generation H1 antihistamines for the long-term management of chronic inducible urticaria (9, 23). Significantly, perioperative opioids can trigger non-immune-mediated histamine release, resulting in a urticarial rash that must be distinguished from true immunological or cholinergic etiologies (20). As patients regain consciousness in the PACU, the sudden onset of rash and pruritus can cause obvious anxiety, especially since this patient had communication difficulties caused by hearing impairment. These emotional fluctuations can further elevate body temperature, perpetuating a vicious cycle. Clinicians must reassure patients, explaining the self - limiting and benign nature of the condition to alleviate distress. After being transferred to the ward, patient and family education should focus on long - term preventive strategies. Specifically, it should involve avoiding known triggers such as strenuous exercise, hot baths, emotional agitation, and high environment temperatures. Postoperative observation following a self-limited cholinergic urticaria episode should be individualized based on symptom severity, recurrence, systemic involvement, and the requirements of the underlying surgical procedure.

Key strengths of this report included the detailed, real-time documentation of core temperature, vital signs, and clinical course throughout the PACU stay, which clearly delineated the temporal relationship between pyrexia and the onset and resolution of cutaneous lesions. In addition, the case systematically excluded perioperative hypersensitivity and provided a pragmatic differential diagnostic framework. It also underscored the importance of obtaining a detailed history of heat- or exercise-induced rash during preanesthesia evaluation, as routine active warming may act as a trigger. When patients are diagnosed with or suspected of having CU, the principle of perioperative temperature management is to maintain normal body temperature without excessive warming. Several limitations should be noted. First, the diagnosis of cholinergic urticaria was based primarily on its characteristic clinical features, the temporal association with the observed rise in core temperature, and the patient's history of exercise- and heat-induced rash; confirmatory provocation or challenge testing was not performed. Second, the patient did not undergo a dermatology consultation, and no systemic treatment for CU was administered.

## 4 Conclusion

Although CU during the post-anesthesia recovery period is infrequently reported, its clinical significance is substantial because of the widespread use of active perioperative temperature management protocols. In this case, the onset was directly precipitated by postoperative shivering, active warming, and extubation stress, which may have driven a rapid elevation in core body temperature. Rigorous differential diagnostic reasoning is imperative. Clinicians must distinguish this benign condition from severe perioperative anaphylaxis by correlating rash morphology, hemodynamic stability, and thermal dynamics. With appropriate symptomatic interventions, judicious use of antihistamines, and careful temperature monitoring, most patients can achieve effective symptom relief while maintaining stable physiological status.

## Data Availability

The original contributions presented in the study are included in the article/Supplementary Material, further inquiries can be directed to the corresponding author.
